# Chromosomal breaks at FRA18C: association with reduced *DOK6* expression, altered oncogenic signaling and increased gastric cancer survival

**DOI:** 10.1038/s41698-017-0012-3

**Published:** 2017-05-01

**Authors:** Siew Hong Leong, Kyaw Myo Lwin, Sze Sing Lee, Wai Har Ng, Kia Min Ng, Soo Yong Tan, Bee Ling Ng, Nigel P. Carter, Carol Tang, Oi Lian Kon

**Affiliations:** 1 Division of Medical Sciences, Humphrey Oei Institute of Cancer Research, National Cancer Centre Singapore, 11 Hospital Drive, Singapore, 169610 Singapore; 2 Department of Biochemistry, National University of Singapore, 8 Medical Drive, Singapore, 117596 Singapore; 3 Department of Pathology, Singapore General Hospital, Outram Road, Singapore, 169608 Singapore; 4 Wellcome Trust Sanger Institute, Wellcome Trust Genome Campus, Hinxton, Cambridge, CB10 1SA UK; 5 National Neuroscience Institute, 11 Jalan Tan Tock Seng, Singapore, 308433 Singapore

## Abstract

Chromosomal rearrangements are common in cancer. More than 50% occur in common fragile sites and disrupt tumor suppressors. However, such rearrangements are not known in gastric cancer. Here we report recurrent 18q2 breakpoints in 6 of 17 gastric cancer cell lines. The rearranged chromosome 18, t(9;18), in MKN7 cells was flow sorted and identified by reverse chromosome painting. High-resolution tiling array hybridization mapped breakpoints to *DOK6* (docking protein 6) intron 4 in FRA18C (18q22.2) and an intergenic region in 9q22.2. The same rearrangement was detected by FISH in 22% of 99 primary gastric cancers. Intron 4 truncation was associated with reduced *DOK6* transcription. Analysis of The Cancer Genome Atlas stomach adenocarcinoma cohort showed significant correlation of *DOK6* expression with histological and molecular phenotypes. Multiple oncogenic signaling pathways (gastrin-CREB, NGF-neurotrophin, PDGF, EGFR, ERK, ERBB4, FGFR1, RAS, VEGFR2 and RAF/MAP kinase) known to be active in aggressive gastric cancers were strikingly diminished in gastric cancers with low *DOK6* expression. Median survival of patients with low *DOK6*-expressing tumors was 2100 days compared with 533 days in patients with high *DOK6*-expressing tumors (log-rank *P* = 0.0027). The level of *DOK6* expression in tumors predicted patient survival independent of TNM stage. These findings point to new functions of human *DOK6* as an adaptor that interacts with diverse molecular components of signaling pathways. Our data suggest that *DOK6* expression is an integrated biomarker of multiple oncogenic signals in gastric cancer and identify FRA18C as a new cancer-associated fragile site.

## Gastric cancer: Chromosome rearrangement linked to less aggressive tumors

Gastric tumors often harbor a chromosome abnormality that disrupts a key signaling gene, resulting in less aggressive cancers. Oi Lian Kon from the National Cancer Centre Singapore and colleagues searched for chromosomal rearrangements in 17 gastric cancer cell lines. The researchers discovered a recurrent breakpoint in six of the cell lines that mapped to a gene called *DOK6*, which encodes a protein that provides a docking platform for multiple signaling molecules. They also found the abnormality in 22 of 99 tissue samples taken from gastric cancer patients. This defect led to lower expression of *DOK6* and, in turn, less active oncogenic signaling pathways. Patients with low *DOK6*-expressing gastric cancers lived longer on average than those with high *DOK6*-expressing tumors. The findings point to *DOK6* levels as a potential drug target and diagnostic biomarker.

## Introduction

Common fragile sites are often involved in chromosomal instability and are more likely to be oncogenic due to a higher content of protein-coding genes and miRNAs than non-fragile sites.^1, 2^ More than 50% of recurrent deletions in cancer genomes map to common fragile sites with large genes.^3^ The best characterized are FRA3B and FRA16D deletions that cause loss of tumor suppressors, fragile histidine triad (*FHIT*) and WW domain containing oxidoreductase (*WWOX*), respectively.^4, 5^ Other common fragile site cancer-associated genes are *PARK2* (Parkin RBR E3 ubiquitin protein ligase) (FRA6E), RAR-related orphan receptor A (*RORA*) (FRA15A), glutamate ionotropic receptor delta type subunit 2 (*GRID2*) (FRA4G), catenin alpha 3 (*CTNNA3*) (FRA10D) and Neurobeachin (*NBEA*) (FRA13A).^6^ Common fragile site instability also occurs in precancerous lesions, probably due to intrinsic sensitivity to replication stress.^7^

FRA18C was first identified in a child with Beckwith–Wiedemann syndrome, and her father.^8^ Both had a pure truncation of 18q22-qter, which disrupted *DOK6* in intron 4. Hemizygous deletion of FRA18C was found among 746 human cancer cell lines derived from 31 different tumor types.^9^ Copy number loss at FRA18C was observed in Barrett’s esophagus.^10^ However, FRA18C deletion has not been identified in gastric cancer.

DOK proteins are adaptors with multiple docking sites for signaling proteins.^11^ They are phosphorylated by protein tyrosine kinases (e.g., Abl Proto-oncogene 1 (Abl), epidermal growth factor receptor (EGFR), platelet-derived growth factor receptor (PDGFR), Kit (KIT proto-oncogene), Src (SRC proto-oncogene) and Tec (Tec protein tyrosine kinase)) and tend to negatively regulate tyrosine kinase signaling networks. Docking protein 1–3 (Dok1–3) are cell-autonomous lung tumor suppressors in mice. Human lung adenocarcinomas show a high frequency of genome copy number loss of docking protein 2 (*DOK2*) associated with decreased *DOK2* transcription.^12^ *DOK2* is also a putative tumor suppressor in human natural killer cell lymphomas.^13^

DOK6 belongs to a different subfamily than DOK1–3. Its C terminus is tyrosine-phosphorylated by Ret (Ret Proto-oncogene)-Src signaling and neurotrophic receptor tyrosine kinase 3 (Ntrk3).^14, 15^ Phosphorylated Dok6 binds phosphorylated Ret and Ntrk3, and mediates neurite outgrowth. Although the functions of human DOK6 are largely unknown,^16^ a possible role in transducing mitogenic and cell proliferation signals is suggested by its binding to EGFR and ERBB2 (Erb-B2 receptor tyrosine kinase 2).^17^ Murine Dok6 is expressed mainly in the brain, nervous tissues and fetal ureteric buds while human *DOK6* is expressed more widely (www.gtexportal.org/home/gene/DOK6; www.proteinatlas.org).

Gene deletions in common fragile sites have been rarely reported in gastric cancer, neither is *DOK6* expression known to have biological associations with gastric cancer. In this study, we report recurrent 18q2 breakpoints affecting 6 of 17 gastric cancer cell lines. The breakpoint mapped to intron 4 of *DOK6* within FRA18C in a gastric cancer cell line, which had greatly reduced *DOK6* transcription. We showed a similar abnormality in 99 primary gastric cancer tissues by FISH. In The Cancer Genome Atlas gastric adenocarcinoma study (TCGA STAD),^18^ *DOK6* expression correlated significantly with gastric cancer phenotypes. Patients with low *DOK6*-expressing gastric cancers survived significantly longer. Analysis of genes that were differentially expressed between low and high *DOK6*-expressing gastric cancers point to a role for *DOK6* in concurrently mediating multiple oncogenic signaling pathways.

## Results

### Recurrent 18q2 rearrangements in gastric cancer cell lines

Spectral karyotyping of 17 gastric cancer cell lines (AGS, Hs 746T, KATO III, NCI-N87, SNU-1, SNU-5, SNU-16, FU97, IM95, MKN7, YCC1, YCC2, YCC3, YCC6, YCC9, YCC11 and YCC16) showed recurrent 18q2 breakpoints in six cell lines (Supplementary Fig. S1). Each cell line showed the characteristic breakpoint in at least 30% of metaphases examined. We focused on MKN7, a near-triploid cell line, which had two copies of t(9;18).

### Breakpoint mapping of 18q2 rearranged chromosome

**Fig. 1:**
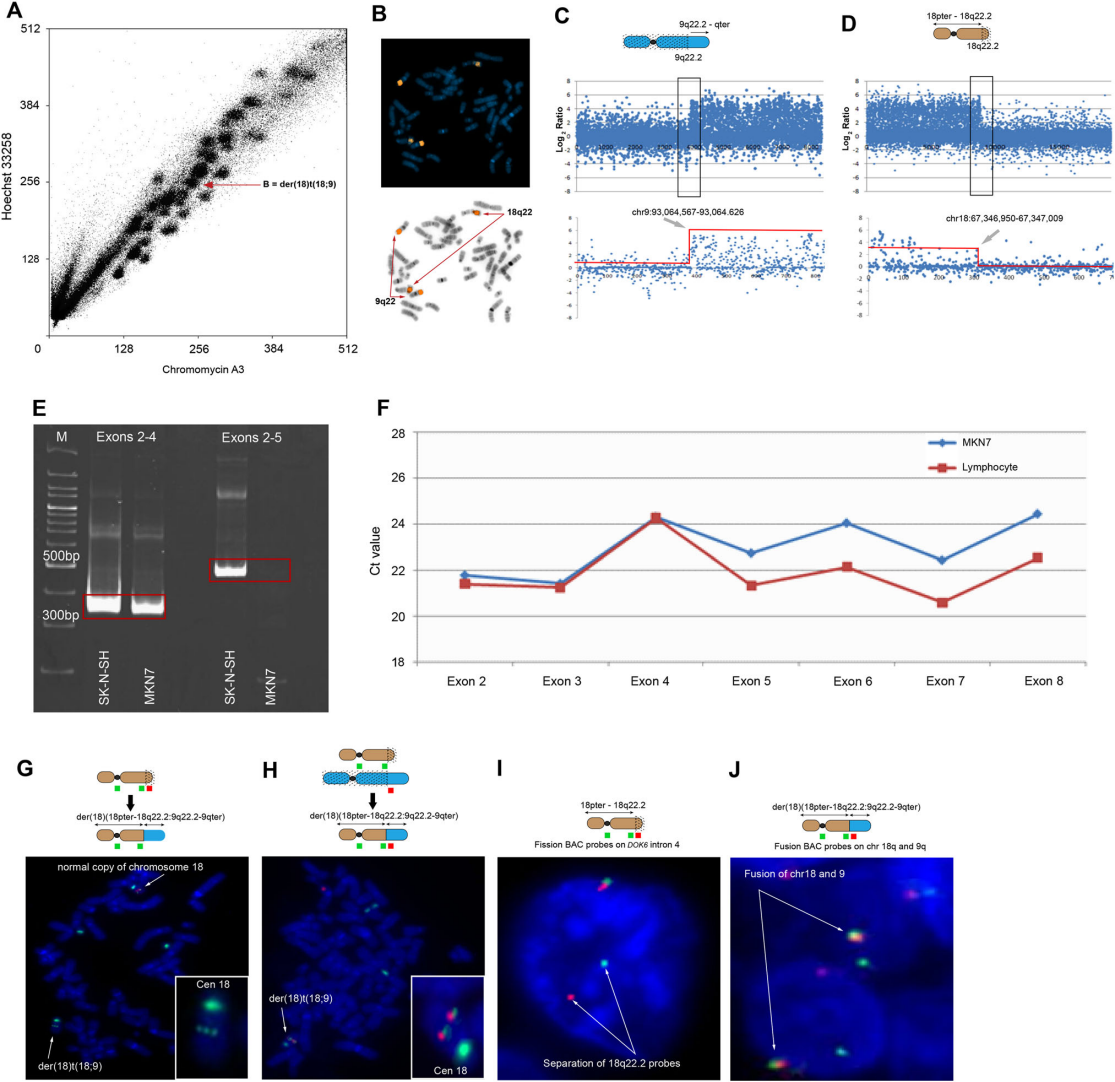
Fine mapping and FISH assays of der(18)t(9;18) **a** Flow karyogram of MKN7 cells. **b** Reverse chromosome painting of spot B. **c**–**d** MKN7 9q2 and 18q2 breakpoints mapped on tiling arrays. Arrows indicate copy number transition points. **e** DOK6 exon-specific RT-PCR of SK-N-SH and MKN7 cDNA. Expression of DOK6 mRNA was determined in two exonic segments: exons 2–4 and exons 2–5. Both exonic segments of correct predicted sizes were expressed in SK-N-SH cells while MKN7 cells expressed only exons 2–4. **f** Exon-specific real-time genomic PCR of individual DOK6 exons (2–8) of MKN7 cells and normal human lymphocytes. C_t_ values diverge after exon 4 and are uniformly higher for MKN7. Primer sequences for (**e**) and (**f**) are in Supplementary Table S1. **g** Fission FISH assay of MKN7 shows loss of red probe (G248P800714D5; telomeric of 18q22 breakpoint) in der(18). Inset: retention of the green probe (G248P89647A7; centromeric of breakpoint) in der(18). Both probes hybridized to normal chromosome 18. **h** Fusion FISH assay of MKN7 shows co-hybridization of probes for 18q22 (G248P89647A7; *green*) and 9q22 (G248P889245F1; *red*). Both insets show a strong green signal for chromosome 18 centromere. **i** Fission FISH assay on primary gastric cancer showing separation of 18q22 probes. **j** Fusion FISH assay on primary gastric cancer showing co-hybridization of 18q22 and 9q22 probes. Details of all FISH probes are in Supplementary Table S6

We flow-sorted MKN7 chromosomes by gating distinct spots in the flow karyogram (Fig. 1a). Spot B was identified as the translocated derivative chromosome 18, der(18)t(9;18), by reverse chromosome painting^19^ (Fig. 1b).

We designed a high-resolution custom oligonucleotide tiling array to focus on the chromosomal regions of interest to define the breakpoints in der(18). Design of the 60-mer oligonucleotide array (Agilent) was guided by approximate genome positions derived from genome copy number data of spot B hybridized on GeneChip® Human Mapping 500K Array Sets (Affymetrix). Probe ends in the custom array were spaced 150 bp apart, giving a tiling resolution of 210 bp. The array had 27,383 unique probes to resolve breakpoints in 18q and 9q, and included 2000 positive and negative control probes. We identified the breakpoints in der(18) to an intergenic region of 9q22.2 (93,064,626 bp) and intron 4 of *DOK6* in 18q22.2 (67,347,009 bp) (Figs. 1c, d). (Base positions refer to GRCh37/hg19.)

### Confirming *DOK6* truncation in 18q22.2

We confirmed truncation of *DOK6* by reverse transcriptase-PCR (RT-PCR) of its transcript and by quantitative PCR of genomic exons using *DOK6* exon-specific primers (primer sequences in Supplementary Table S1). RT-PCR of MKN7 RNA with primers spanning exons 2–4 amplified a product of the correct predicted size but RT-PCR spanning exons 2–5 was negative. In contrast, RT-PCR of RNA from SK-N-SH cells, which express full length *DOK6*,^16^ amplified both exon 2–4 and exon 2–5 transcripts of correct predicted sizes (Fig. 1e). Thus, the 18q22.2 breakpoint abolished transcription of *DOK6* mRNA downstream of exon 4. As further confirmation, quantitative PCR on single *DOK6* exons in MKN and normal human lymphocyte genomic DNA using exon-specific primers showed comparable C_t_ values for exons 2, 3 and 4 in both MKN7 cells and normal lymphocytes. However, C_t_ values for exons 5, 6, 7 and 8 were uniformly higher in MKN genomic DNA consistent with deletion 3ʹ of intron 4, (Fig. 1f). Thus, analyses of mRNA and genomic DNA were concordant in confirming truncation of *DOK6* within intron 4.

### Fosmid FISH

We validated breakpoints mapped from the custom array by fosmid FISH on MKN7 cell metaphases. Fission FISH assay using a pair of fosmids flanking the intron 4 breakpoint (G248P89647A7 on chr18:67,284,306–67,321,278 labeled with SpectrumGreen; and G248P800714D5 on chr18:67,362,172–67,398,114 labeled with SpectrumOrange) showed loss of the telomeric probe (G248P800714D5) and retention of the upstream green probe (G248P89647A7), confirming the breakpoint within *DOK6* intron 4 (Fig. 1g). To demonstrate fusion of truncated *DOK6* with chromosome 9q22.2 in der(18), we co-hybridized a chromosome 9 fosmid probe (G248P88925F1; chr9:93,055,252–93,095,045 labeled with SpectrumOrange) with G248P89647A7 on MKN7 metaphases. Admixture of green and red signals showed fusion between chromosomes 18q22.2 and 9q22.2 (Fig. 1h).

### Frequency of der(18)t(9;18) in primary gastric cancer

As cancer cell lines retain features of their cognate primary tumor types, we used breakpoint localization coordinates to determine whether the translocation in MKN7, t(9;18)(qter→q22.2::q22.2→pter), was also present in primary gastric cancer tissues. Breakpoint positions identified from the custom array were used to select BAC probes that overlapped the loci of the fosmid probes. We screened 99 primary gastric cancer tissues (Supplementary Table S2) and found the same rearranged chromosome, t(9;18)(q22.2;q22.2), in 22 tumors. In a typical gastric cancer harboring this abnormal chromosome, truncation of *DOK6* intron 4 was evident with break-apart BAC probes flanking the breakpoint (RP11-22G18; chr18:67,564,184–67,743,728 labeled with SpectrumGreen and RP11-98M3; chr18: 67,198,763–67,355,215 labeled with SpectrumOrange) (Fig. 1i). Fusion between chromosomes 18 and 9 in this tumor was shown by co-hybridizing RP11-98M3 (labeled with SpectrumOrange) and RP11-133K16 (chr9:93,540,070–93,720,725 labeled with SpectrumGreen) (Fig. 1j).

### *DOK6* expression in TCGA gastric cancers

Our data indicated that der(18) would not generate a fusion protein since the first four exons of *DOK6* were fused to a non-coding intergenic region of chromosome 9. We reasoned that the breakpoint would instead reduce *DOK6* expression by nonsense-mediated RNA decay. This was supported by transcriptome analysis that showed >13-fold reduction in *DOK6* expression in MKN7 cells compared to normal stomach tissues. We analyzed data from the TCGA STAD study^18^ to investigate correlations of *DOK6* expression with histological and molecular gastric cancer phenotypes. There was a wide range of *DOK6* expression among TCGA STAD tumors. The mean RPKM value in the highest quartile of *DOK6* expression (*n* = 96) was 6.4 times higher than the corresponding mean in the lowest quartile (*n* = 96) (*P* = 1.85 × 10^−48^; Welch’s *t*-test). Genome copy number deletion at FRA18C was significantly more frequent among gastric cancers in the lowest quartile of *DOK6* expression (24 of 96 tumors) compared to the highest quartile (9 of 96 tumors) (*P* = 0.0041; Fisher’s exact test). This was consistent with deletion at the FRA18C locus as one cause of reduced *DOK6* transcription. If true, the expression of 18q22.2 genes telomeric to *DOK6* would also be reduced. We compared the expression of 34 genes telomeric to *DOK6* for which RPKM values were available between two gastric cancer groups in the TCGA STAD data set. The first group comprised gastric cancers that had low *DOK6* expression and FRA18C copy number deletion (*n* = 24); the second group comprised all other gastric cancers in the same data set without FRA18C deletion and *DOK6* expression above the lowest quartile (*n* = 229). Table 1 shows reduced expression of all 34 genes except LOC400655, of which 28 genes were significantly underexpressed. *DOK6* expression itself was more than 3.8-fold reduced, consistent with its deletion within the FRA18C locus. Taken together, copy number and transcriptome analysis identified a subset of primary gastric cancers with a chromosomal break at FRA18C characterized by diminished *DOK6* expression.

**Table 1:** Reduced transcription of genes telomeric to *DOK6* in gastric cancer with FRA18C copy number deletion

Gene ID	−Fold decrease (RPKM)	*P* (adjusted for FDR 0.05)
ADNP2*	−1.133	1.24 × 10^−2^
ATP9B*	−1.263	3.63 × 10^−4^
C18orf63	−3.636	6.33 × 10^−2^
CBLN2*	−2.047	1.24 × 10^−2^
CD226*	−1.864	2.86 × 10^−4^
CNDP1*	−2.439	7.58 × 10^−4^
CNDP2*	−1.104	1.44 × 10^−3^
CTDP1*	−1.167	2.67 × 10^−6^
CYB5A	−1.040	1.13 × 10^−1^
DOK6*	−3.759	1.03 × 10^−47^
FAM69C*	−1.943	4.55 × 10^−3^
FBXO15*	−2.093	9.76 × 10^−3^
GALR1*	−4.221	1.77 × 10^−4^
HSBP1L1	−1.006	8.51 × 10^−1^
KCNG2*	−1.960	6.50 × 10^−7^
LOC100131655*	−1.602	3.54 × 10^−5^
LOC100505817*	−7.015	4.64 × 10^−4^
LOC339298	−2.698	1.14 × 10^−1^
LOC400655	+0.814	6.98 × 10^−1^
MBP*	−1.335	3.40 × 10^−7^
NETO1	−2.313	5.68 × 10^−2^
NFATC1*	−1.471	1.37 × 10^−5^
PARD6G*	−1.600	4.99 × 10^−5^
PQLC1*	−1.148	1.01 × 10^−6^
RBFA*	−1.229	1.72 × 10^−4^
RTTN*	−1.272	2.22 × 10^−3^
SALL3*	−6.319	7.57 × 10^−5^
SOCS6*	−1.154	2.51 × 10^−3^
TIMM21*	−1.091	2.91 × 10^−3^
TSHZ1*	−1.406	5.93 × 10^−7^
TXNL4A	−1.043	5.68 × 10^−2^
ZADH2*	−1.263	1.06 × 10^−7^
ZNF236*	−1.303	6.50 × 10^−7^
ZNF407*	−1.416	1.67 × 10^−8^
ZNF516*	−1.454	1.83 × 10^−5^

Asterisks denote significantly reduced transcription compared to gastric cancers without FRA18C deletion by Welch’s *t*-test

**Fig. 2:**
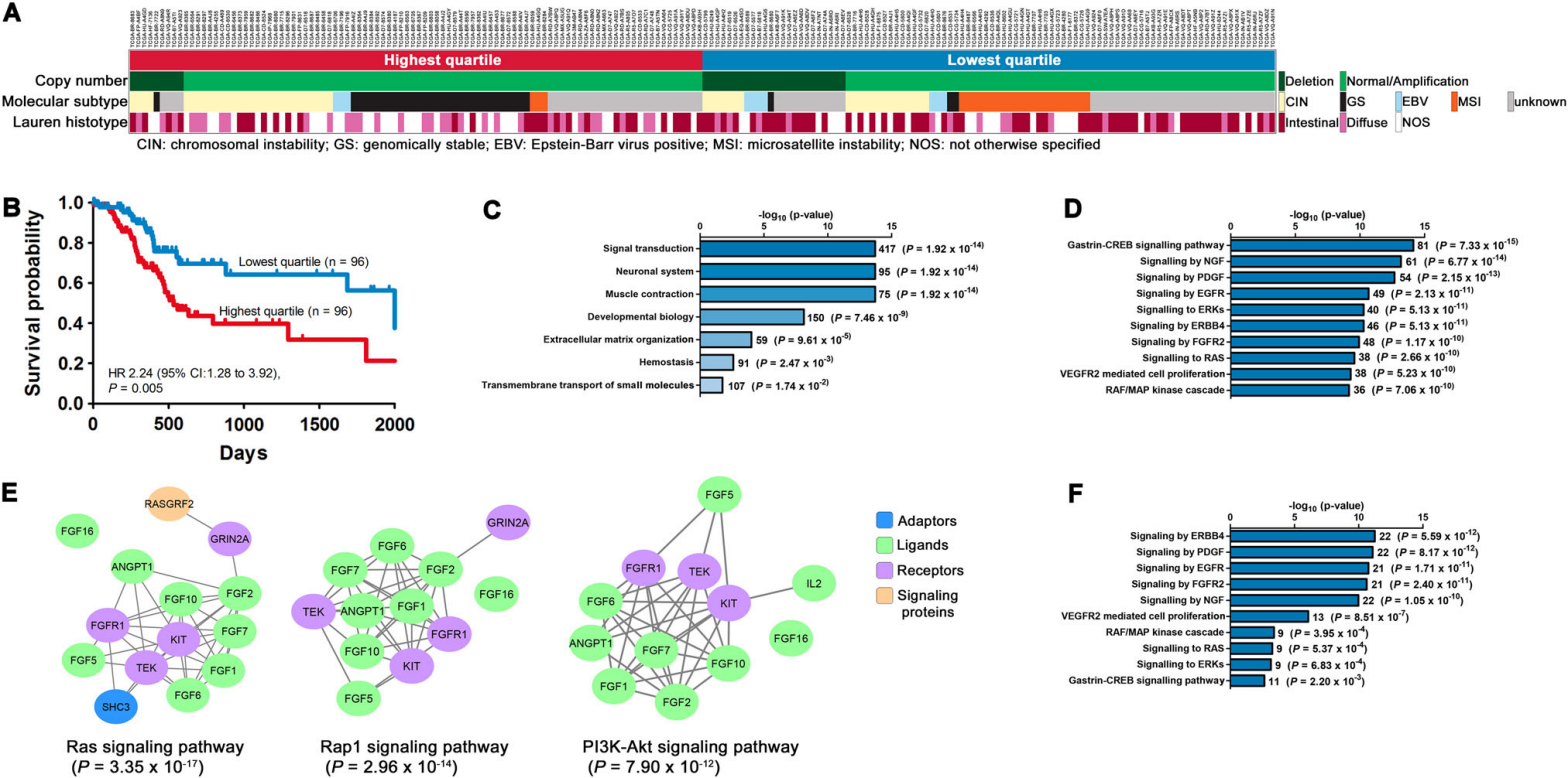
Clinicopathological correlations of gastric cancers with *DOK6* expression. **a** Association of the highest and lowest quartiles of *DOK6*-expressing gastric cancers with FRA18C copy number status, Lauren histotype and molecular subtype. Columns represent individual gastric cancer tumors of the respective quartiles. **b** Kaplan–Meier survival plot showing longer survival of patients in the lowest quartile. Data for (**a**) and (**b**) are from the TCGA STAD study. **c** Processes mapped by Reactome for genes underexpressed in the lowest compared to the highest quartile. **d** Top 10 canonical pathways represented by 417 genes of the signal transduction process in (**c**). **e** Signaling pathways enriched in 34 core genes present in all 10 pathways in (**d**) by STRING analysis. **f** Signaling pathways enriched in differentially expressed proteins (RPPA data). The number of genes and FDR-corrected *P*-values are shown for each bar in (**c**, **d** and **f)**

*DOK6* expression correlated with several histopathological, molecular and clinical features of gastric cancer tumors. A majority of gastric cancers in the lowest quartile (55 of 96) were of the Lauren intestinal histotype, whereas gastric cancers in the highest quartile were mainly of the diffuse histotype (31 of 95) (Pearson χ^2^ = 15.956, *P* = 3.43 × 10^−4^). The microsatellite unstable molecular subtype was more prevalent among the lowest quartile gastric cancers (22 of 53) while gastric cancers in the highest quartile were mainly of the genomically stable subtype (31 of 66) (Pearson χ^2^ = 39.429, *P* = 1 × 10^−8^) (Fig. 2a). In the TCGA STAD cohort, patients whose tumors were in the lowest quartile of *DOK6* expression survived significantly longer (median survival 2100 days) than patients whose tumors were in the highest quartile (median survival 533 days) (log-rank *P* = 0.0027; Fig. 2b). *DOK6* expression predicted patient survival independent of age, gender, histotype and TNM stage by Cox regression analysis. The risk of death in the highest quartile was more than twice to that in the lowest quartile (hazard ratio 2.24; confidence interval 1.28–3.92; *P* = 0.005)

To elucidate the possible role of *DOK6* in gastric carcinogenesis, analysis of differentially expressed genes (≥2-fold different) revealed 3797 genes were underexpressed and 37 genes were overexpressed in the lowest quartile compared to the highest quartile gastric cancers. Using the Reactome knowledge base,^20^ the underexpressed genes mapped significantly to seven biological processes, of which signal transduction strikingly emerged as the single process with the largest number of mapped genes (417 genes) (Fig. 2c). This marked effect on signaling was consistent with the loss of the phosphotyrosine-binding domain encoded by exon 5 of *DOK6* in the lowest quartile gastric cancers.

Analysis with Reactome software of 417 signal transduction genes that were differentially underexpressed between gastric cancers in the lowest and highest quartile mapped to multiple signaling pathways. The ten most significant pathways were signaling by gastrin-CAMP responsive element-binding protein (CREB) (*P* = 7.33 × 10^−15^), Neurotrophin (NGF) (*P* = 6.77 × 10^−14^), PDGF (*P* = 2.15 × 10^−13^), EGFR (*P* = 2.13 × 10^−11^), ERKs (mitogen-activated protein kinases) (*P* = 5.13 × 10^−11^), Erb-B2 receptor tyrosine kinase 4 (ERBB4) (*P* = 5.13 × 10^−11^), fibroblast growth factor receptor 2 (FGFR2) *P* = 1.17 × 10^−10^), Ras Proto-oncogene (RAS) (*P* = 2.66 × 10^−10^), vascular endothelial growth factor receptor 2 (VEGFR2) (*P* = 5.23 × 10^−10^) and Raf proto-oncogene (RAF)/mitogen-activated protein kinase (*P* = 7.06 × 10^−10^) (all *P* values are FDR-adjusted) (Fig. 2d). From the ten gene sets of each pathway, we extracted a core group of 34 genes that were present in all ten pathways. These 34 genes encode proteins in six functional groups: receptors and receptor components (*FGFR1*, GDNF family receptor alpha 1 (*GFRA1*), *GFRA2*, *GFRA4*, glutamate ionotropic receptor NMDA-type subunit 2A (*GRIN2A*), interleukin 17 receptor D (*IL17RD*), IL 5 receptor subunit alpha (*IL5RA*), integrin subunit beta 3 (*ITGB3*), *KIT*, *RET* and TEK receptor tyrosine kinase (*TEK*)); ligands (angiopoietin 1 (*ANGPT1*), *FGF1*, *FGF10*, *FGF16*, *FGF2*, *FGF5*, *FGF6*, *FGF7*, glial cell-derived neurotrophic factor (*GDNF*), *IL2*, *IL5*, neuregulin 2 (*NRG2*) and *NRG3*); signaling proteins (calcium/calmodulin-dependent protein kinase II alpha (*CAMK2A*) and Ras protein-specific guanine nucleotide releasing factor 2 (*RASGRF2*)); signaling adaptors (connector enhancer of kinase suppressor of Ras 2 (*CNKSR2*), Klotho (*KL*) and SHC adaptor protein 3 (*SHC3*)); cell–cell interactors (neural cell adhesion molecule 1 (*NCAM1*); and cytoskeleton proteins (actinin alpha 2 (*ACTN2*), neurofilament, light polypeptide (*NEFL*), spectrin alpha, erythrocytic 1 (*SPTA1*) and spectrin beta, non-erythrocytic 2 (*SPTBN2*)). As *DOK6* encodes a signaling adaptor protein, we investigated the relationship between its expression and each of the 34 genes. Using Spearman correlation analysis on TCGA STAD transcriptome data of all gastric cancer patients (*n* = 384), there was significant co-expression of *DOK6* with each of the 34 genes (Supplementary Table S3). Among this core set of 34 genes, *DOK6* was most strongly co-expressed with five ligands (*ANGPT1*, *FGF1*, *FGF2*, *FGF7* and *FGF10*), three receptors (*FGFR1*, *ITGB3* and *TEK*), two adaptors (*CNKSR2* and *KL*) and a signaling protein (*RASGRF2*) (*ϒ*_S_ > 0.60). We used the STRING v10 database (string-db.org; confidence score 0.7) to determine functional interactions among the 34 genes.^21^ This showed highly significant enrichment of three Kyoto Encyclopedia of Genes and Genomes (KEGG)^22^ signaling pathways: Ras (FDR *P* = 3.35 × 10^−17^); Rap1 (RAP1 GTPase activating protein) (FDR *P* = 2.96 × 10^−14^) and PI3K (phosphatidylinositol-4,5-bisphosphate 3-kinase)-Akt (AKT serine/threonine kinase) (FDR *P* = 7.90 × 10^−12^) (Fig. 2e).

Analysis of reverse phase protein array (RPPA) data from TCGA STAD showed 60 proteins whose expressions were significantly different between the lowest and highest quartile gastric cancers (Supplementary Table S4). Reactome analysis of these differentially expressed proteins confirmed enrichment of the same signaling pathways identified from transcriptome analysis: ERBB4 (*P* = 5.59 × 10^−12^), PDGF (*P* = 8.17 × 10^−12^), EGFR (*P* = 1.71 × 10^−11^), FGFR2 (*P* = 2.40 × 10^−11^), NGF (*P* = 1.05 × 10^−10^) and VEGFR2-mediated cell proliferation (*P* = 8.51 × 10^−7^) (Fig. 2f and Supplementary Fig. S2).

## Discussion

By combining chromosome sorting, array painting and high-resolution copy number analysis, we identified recurrent truncation of *DOK6* in intron 4 at the FRA18C locus in MKN7 cells and found the same abnormality in 22% of primary gastric cancer tumors by break-apart FISH assay. *DOK6* truncation is consistent with mainly intragenic chromosomal rearrangements and frequent 18q copy number loss in gastric cancer.^23^ The frequency of 18q22.2 breaks in this study was higher than that of other chromosomal rearrangements previously reported in gastric cancer and establishes FRA18C as a new cancer-associated common fragile site. Our results further suggest that the resulting decrease in *DOK6* expression is not a bystander abnormality, as there were several significant molecular and clinical differences between the lowest and highest quartiles of *DOK6*-expressing gastric cancers, notably significantly longer survival associated with low DOK6 expression. Copy number loss at FRA18C was significantly more prevalent but not uniform among gastric cancers in the lowest quartile, indicating that *DOK6* expression was reduced in some tumors by mechanisms other than copy number loss.

A striking majority of differentially expressed genes in low compared to high *DOK6*-expressing gastric cancers were underexpressed (3797 of 3834 genes; 99%), among which the largest functional group were genes involved in signal transduction. These mapped with high statistical significance to ten canonical signaling pathways, all of which were less active in low *DOK6* gastric cancers: gastrin-CREB, NGF, PDGF, EGFR, ERKs, ERBB4, FGFR2, RAS, VEGFR2 and RAF/MAP kinase. A core group of 34 genes present in all ten pathways were readily assigned to functional classes involved in different steps in signal transduction, i.e., receptors and receptor components, ligands, signaling proteins, signaling adaptors, cell–cell interactors and cytoskeletal proteins. This functional diversity suggests that DOK6 engages with various components at different steps in multiple signaling pathways. Every gene in the core group was significantly co-expressed with *DOK6*, with genes encoding ligands, receptors, adaptors and a signaling protein showing the strongest correlation. This conforms to known characteristics of co-expressed genes which tend to be functionally related, involved in the same biological processes and encode interacting proteins.^24^ To date, DOK6 has been shown to function as an adaptor only in neurite outgrowth, a process in which it mediates *Ret* and neurotrophin-3 signaling.^14, 15^ Our analyses indicate that DOK6 functions much more broadly in several oncogenic signaling pathways in gastric cancer, and provides functional relevance of its binding to EGFR.^17^ The pleiotropic functions of DOK6 accord with estimates that, on average, eukaryotic proteins interact with four to eight proteins; and that each protein can be involved in about ten different biological functions.^25^

Some signaling pathways active in TCGA STAD tumors with high *DOK6* expression are known adverse prognostic factors. Several reports have associated expression of EGFR,^26, 27^ FGFR,^28, 29^ PDGFR^30^ and VEGR,^31^ and their respective ligands in gastric cancer with more aggressive tumors and poorer survival. Although neurotrophins are best known for their effects on neurons and tumors of the nervous system, they also exert effects on tumors outside the nervous system.^32^ Neurotrophin signaling has been implicated in leukemogenesis,^33^ and is a prognostic factor in human pancreatic and colorectal cancer.^34, 35^ It is plausible that DOK6 mediates neurotrophin/NGF signaling because DOK5, which occupies the same phylogenetic branch of the DOK family, functions as an adaptor in neurotrophin-3/TrkC signaling.^36^

Our study has shown that high expression of a single adaptor protein, DOK6, is associated with increased activity of multiple oncogenic pathways, implying that this multi-site docking protein enhances many signaling pathways by interacting with a diverse range of different receptors and signaling proteins. Through its N-terminal pleckstrin homology domain, DOK6 recruits signal transducing proteins to membranes. Dok6 itself can be tyrosine-phosphorylated at its C terminus, thus facilitating docking and assembly of downstream effector proteins in large signaling complexes. Its IRS-type phosphotyrosine-binding domain interacts with phosphorylated Ret, and potentially could interact with other oncogenic kinases, e.g., RET, EGFR and ERBB2. Stimulation of oncogenic signaling by high *DOK6* expression is further augmented by cross-talk among these pathways, e.g., gastrin-EGFR cross talk. EGFR activity stimulates gastrin gene transcription while gastrin increases the expression of EGFR ligands.^37, 38^ The significantly poorer survival of gastrin-positive gastric cancers may in part reflect such augmented oncogenic signaling.^39^ Viewed in this manner, *DOK6* expression may be regarded as an integrated modulator for the concurrent activities of multiple oncogenic signaling pathways. Their combined oncogenic effects could explain the significantly shorter survival of gastric cancer patients with high *DOK6* tumors (Fig. 2b) and explain why the level of *DOK6* expression alone is prognostic independent of TNM stage, currently the only prognosticator in clinical practice.

Results of this study are relevant to the phenomenon of resistance to targeted therapy. Although trastuzumab is an approved therapy for gastric cancers with *ERBB2* overexpression or amplification, clinical response rates and improvements in median overall survival are short-lived and modest. Moreover, the majority of *ERBB2*-positive gastric cancers do not respond to trastuzumab therapy, indicating that inhibition of *ERBB2* signaling alone is either ineffective or only transiently effective.^40^ Our observations show that multiple signaling pathways operate in parallel in gastric cancer. This is in accord with a report that most *ERBB2*-positive gastric cancers have several additional co-existing abnormalities such as focal amplifications and somatic mutations of other oncogenes and cell cycle genes. These accompanying alterations manifested as cell cycle dysregulation and activated oncogenic kinase signaling. Combination targeted therapy that simultaneously inhibited more than one signaling pathway overcame intrinsic resistance to trastuzumab, demonstrating that the cancer phenotype in general is sustained by more than a single oncogenic pathway or process.^41^ Our data show that DOK6 has a key role in supporting cancer development and growth, and suggest that combination therapy from the outset to inhibit intersecting pathways and processes, rather than sequential monotherapy, may improve therapeutic outcomes for gastric cancer patients. This is supported by data from two pre-clinical studies that showed the superior anti-proliferative effects of combinations of targeted agents in gastric cancer cell lines and patient-derived gastric cancer xenotumors compared to monotherapy. A pan-HER inhibitor, PF0029984, was tested in seven gastric cancer cell lines, each having one of the following abnormalities: *KRAS* mutation; amplification of *FGFR2*, *MYC* (V-Myc avian myelocytomatosis viral oncogene homolog) or *MET* (MET proto-oncogene receptor tyrosine kinase). Combining PF0029984 or trastuzumab with another targeted agent (inhibitor of insulin-like growth factor 1 receptor, ERK1/2 or PI3K/mechanistic target of rapamycin) appropriate to the genotype of the cell line tested resulted in synergistic cytotoxic effects in most cases.^42^ In NCI-N87 xenograft tumors, combined treatment with trastuzumab and VEGF-Trap inhibited tumor growth more effectively than monotherapy with either agent.^43^ Results of a recent clinical trial also point to superiority of combination therapy. A single-arm trial of advanced HER2-positive gastric cancer patients treated with chemotherapy and two targeted agents (trastuzumab and bevacizumab) reported longer progression-free survival than the ToGA trial, which treated patients with chemotherapy and trastuzumab only.^44^

## Methods

### Cell cultures

Seven gastric cancer cell lines (AGS, Hs746T, KATO III, NCI-N87, SNU-1, SNU-5, SNU-16) were purchased from the American Type Culture Collection (Manassas, VA, USA) and three (FU97, IM95, MKN7) from Japanese Collection of Research Bioresources (Osaka, Japan). YCC1, YCC2, YCC3, YCC6, YCC9, YCC11, YCC16 cell lines were derived from gastric cancer patients at the Yonsei Cancer Center, Seoul, Korea and were a kind gift from Professor S.Y. Rha. All cell lines were cultured in conditions recommended by their respective sources. SK-N-SH, a human neuroblastoma cell line that expresses full length *DOK6*, was from the American Type Culture Collection and cultured in MEM with 10% fetal bovine serum.

### Genomic DNA extraction

MKN7 and normal human lymphocyte genomic DNA were isolated using Blood and Cell Culture DNA Midi kit and the manufacturer’s protocol (Qiagen GmbH, Hilden, Germany).

### Spectral karyotyping

Metaphase spreads were prepared using standard protocols. MKN7 cells were treated with colcemid (0.1μg/ml) for 3–6 h, followed by 0.075 M KCl to induce swelling. Cells were fixed in methanol:acetic acid (3:1; v/v) and dropped onto glass slides. Spectral karyotype paint assay was performed as in the manufacturer’s protocol (Applied Spectral Imaging, Neckarhausen, Germany). Breakpoints were considered recurrent if they occurred in at least 3 of 10 metaphases and in 3 or more cell lines.

### Chromosome preparation and sorting

Chromosomes in suspension were prepared from MKN7 cells, and stained with chromomycin A3 and Hoechst 33258. Der(18)t(18;9), chromosomes 1 and 2 were purified by sorting stained chromosomes on a MoFlo® flow cytometer (Beckman Coulter, Inc. Indianapolis, Indiana, USA) as described.^45^ The identity and purity of sorted chromosomes were confirmed by reverse chromosome painting on normal human metaphases.^19^

### Custom tiling array

We used eArray (https://earray.chem.agilent.com/earray) to select 60-mer probes mapping to breakpoint regions (human genome hg19 version) for printing, with a uniform end-to-end distance of 150 bp between consecutive probes (Agilent Technologies, Santa Clara, CA, USA). The custom array had 8335 probes for chromosome 9 and 19,048 probes for chromosome 18. Additional probes served as Agilent control grid features (6539 probes), 1000 normalization probes (chromosome 2), 5000 replicate probes (chromosome 2) to determine variance, 1400 user-defined positive probes (chromosomes 1p, 9p and 18q) and 600 user-defined negative probes (chromosomes 4p and 7p) **(**Supplementary Table S5). The custom array design is accessible at www.ebi.ac.uk/arrayexpress (accession number A-MEXP-2071).

DNA from 1000 copies each of der(18)t(9;18), chromosomes 1 and 2 was amplified by DOP PCR. Der(18)t(9;18) DNA (475 ng) spiked with 25 ng chromosome 2 DNA (for normalization) was labeled with cyanine-5-dUTP. Chromosome 1 DNA spiked with 25 ng chromosome 2 was labeled with cyanine-3-dUTP. All labeling was performed using the Universal Linkage System (Kreatech Biotechnology B.V., Amsterdam, Netherlands). Hybridization of labeled DNA samples to the custom tiling array was performed according to the supplier’s oligonucleotide array-based CGH protocol (Agilent). As the array had a dual-channel design, we co-hybridized der(18) DNA with chromosome 1 DNA to avoid competition for probes in the breakpoint regions. Five replicate sets of 1000 chromosome 2 probes were used to calculate the coefficient of variance. A sixth set of the same chromosome 2 probes corrected for dye bias. Raw signal intensities from the scanned arrays were analyzed by the Feature Extraction software to generate processed signal intensity values. Each probe signal was normalized for background subtraction, spatial detrending as in slide spatial correction and backbone control correction before aberrations were called and log_2_ ratio calculated by Genomic Workbench software (Agilent). The quality of data was shown by reproducibility metrics. Using standards developed by the International Standards for Cytogenomic Arrays Consortium, we set Aberration Detection Module-1 (ADM-1) algorithm with a threshold of 6.7, and required at least four consecutive probes for an aberration call.

### Reverse transcriptase-PCR

First-strand cDNA was synthesized using 200U SuperScript III RT (Invitrogen Life Technologies, Carlsbad, CA, USA) from total RNA (5 µg) of SK-N-SH and MKN7 cells and 50 µM oligo (dT)_18_ in buffer containing 5 mM DTT, 0.5 mM dNTP mix and 20 units RNasin Ribonuclease Inhibitor (Promega) at 50 °C for 1 h. RNA was removed by adding 2U *Escherichia coli* RNase H (Invitrogen Life Technologies) at 37 °C for 20 min. *DOK6* gene-specific primers were used for second-strand synthesis and amplification to determine expression of the following exonic regions: (a) exons 2–4; and (b) exons 2–5. All primer sequences are in Supplementary Table S1. The cycling conditions were: pre-denaturation at 94 °C for 3 min, 35 cycles of 1 min at 94 °C, 30 s at 56 °C and 30 s at 72 °C; followed by a final extension at 72 °C for 3 min. Amplified PCR products were analyzed on 6% TBE polyacrylamide gel at 80 V for 65 min.

### Real-time quantitative genomic PCR

We performed real-time PCR to confirm the 18q breakpoint using the QuantiTech SYBR Green PCR kit (Qiagen) on genomic DNA from MKN7 cells and normal human lymphocytes using exon-specific primers pairs to amplify individual exons 2–8 of *DOK6* (Supplementary Table S1). Each exon was amplified in quadruplicate reactions from which the mean and standard deviation of cycle threshold (Ct) values were calculated and compared between MKN7 cells and normal lymphocytes.

### Fosmid FISH

Fosmid clones (Children’s Hospital Oakland Research Institute, Oakland, CA, USA) that mapped to boundaries of the breakpoint on 18q2 as determined by custom tiling array data, were labeled with SpectrumGreen or SpectrumOrange (Vysis/Abbott Molecular, Des Plaines, IL, USA) using the BioPrime® DNA labeling system (Invitrogen). Labeled probes were overlaid on MKN7 metaphase spreads and hybridized overnight at 37 °C. A chromosome 18 centromeric probe was used to identify chromosome 18 and its derivatives. FISH images were digitally captured and analyzed using FISHview imaging software (Applied Spectral Imaging).

### BAC FISH on primary gastric adenocarcinoma tissues

BAC clones (Children’s Hospital Oakland Research Institute) spanning the breakpoints on chromosomes 9 and 18 were labeled as described above and hybridized to 2-μm sections of gastric cancer tissue microarrays using the Histology FISH Accessory Kit and protocol (Dako, Glostrup, Denmark). Tissue microarrays were constructed from formalin-fixed paraffin-embedded primary gastric adenocarcinoma tissues obtained from 99 patients treated at Singapore General Hospital during the period 2005–2008. This study was approved by the Singapore General Hospital Institutional Review Board.

All fosmid and BAC probes were first validated on normal human metaphases before using them in FISH experiments. Details of all fosmid and BAC FISH probes are in Supplementary Table S6.

FISH signals of 50–100 cancer cells from each tumor were enumerated. In tumors harboring t(9;18)(9q22.2;18q22.2), truncation of *DOK6* intron 4 was demonstrated using a pair of break-apart BAC probes flanking the breakpoint (RP11-22G18; chr18:67,564,184–67,743,728 labeled with SpectrumGreen and RP11-98M3; chr18:67,198,763–67,355,215 labeled with SpectrumOrange). Signals were considered to be split when the distance between the dual colors was greater than twice the diameter of the larger signal. The diagnostic threshold for designating tumors as break-apart positive was the presence of split signals in >20% of cancer cells. Fusion between chromosomes 18 and 9 was shown by co-hybridizing RP11-98M3 (labeled with SpectrumOrange) and RP11-133K16 (chr9:93,540,070–93,720,725 labeled with SpectrumGreen).

### Bioinformatic analysis of TCGA STAD (gastric adenocarcinoma) data

#### Patient stratification based on *DOK6* expression

RPKM values (Reads Per Kilobase per Million mapped reads) measured using Illumina Hiseq 2000 RNA sequencing platform of 384 stomach adenocarcinomas were downloaded from UCSC Cancer Browser (genome-cancer.ucsc.edu/proj/site/hgHeatmap; data freeze 23 March 2016). Patients were stratified into equal quartiles based on *DOK6* transcript RPKM values (*n* = 96 in each quartile).

#### TCGA STAD *DOK6* locus copy number data

Segmented copy number data based on hg19 were obtained from the TCGA Data Portal website (https://tcga-data.nci.nih.gov/tcga/; data freeze 15 November 2015). Mean log_2_ copy number ratios of segments that covered the *DOK6* genomic locus were extracted. Mean log_2_ ratios >0.33 and <−0.33 were considered to be amplifications and deletions, respectively.

#### TCGA STAD clinical data

Clinical data were downloaded from UCSC Cancer Browser (data freeze 23 March 2016). Overall survival data of patients in the highest and lowest *DOK6*-expressing gastric cancer quartiles were estimated by the Kaplan–Meier method. Cox proportional hazard models were fitted to estimate hazard ratio and to assess the association of various clinical characteristics with overall survival. Statistical analyses were performed using SAS v9.4 (SAS Institute Inc., Cary, North CA, USA). Differences in categorical characteristics between the highest and lowest quartiles were compared using the χ^2^ test. The highest and lowest quartiles were compared for distribution of Lauren histotypes (intestinal and diffuse), and molecular subtypes (microsatellite unstable and genomically stable) obtained from published data.^18^ All reported *P*-values were two-sided; a value <0.05 was considered statistically significant.

#### Transcriptome analysis

Differentially expressed genes were identified by comparing mean RPKM values of the lowest vs. the highest quartile. Overexpressed transcripts were ≥2-fold higher in the lowest quartile and significantly different by Welch’s *t*-test with FDR correction of 0.05. Underexpressed transcripts were ≥2-fold lower in the lowest quartile and significantly different by the same criterion. Pathways which mapped to differentially expressed genes were identified using the Reactome pathway knowledge base (http://www.reactome.org/).^20^ Enriched pathways were statistically significant with FDR correction of 0.05.

#### RPPA data analysis

Protein expression for TCGA STAD tumors were downloaded from TCGA Data Portal website (https://tcga-data.nci.nih.gov/tcga/; data freeze 23 February 2016). The most updated antibody list was obtained from the MD Anderson RPPA website (https://www.mdanderson.org/education-and-research/resources-for-professionals/scientific-resources/core-facilities-and-services/functional-proteomics-rppa-core/antibody-lists-protocols/functional-proteomics-reverse-phase-protein-array-core-facility-antibody-lists-and-protocols.html; data freeze 16 March 2016). RPPA data were available for 78 and 68 tumors from the highest and lowest *DOK6*-expressing quartiles, respectively. Only protein expression data from validated antibodies were analyzed. Overexpressed proteins were those with significantly stronger protein expression in the lowest quartile compared to the highest quartile by Welch’s *t*-test after FDR correction of 0.05. Underexpressed proteins were those with significantly weaker protein expression in the lowest quartile compared to the highest quartile by the same criterion. Reactome database was used to identify signaling pathways enriched in the differentially expressed proteins.

## Electronic supplementary material

Supplementary Information

## Electronic supplementary material

Supplementary Information accompanies the paper on the *npj Precision Oncology* website (doi:10.1038/s41698-017-0012-3).

## References

[1] Yunis JJ, Soreng AL (1984). Constitutive fragile sites and cancer. Science.

[2] Laganà A (2010). Variability in the incidence of miRNAs and genes in fragile sites and the role of repeats and CpG islands in the distribution of genetic material. PLoS ONE.

[3] Le Tallec B (2013). Common fragile site profiling in epithelial and erythroid cells reveals that most recurrent cancer deletions lie in fragile sites hosting large genes. Cell. Rep..

[4] Ohta M (1996). The FHIT gene, spanning the chromosome 3p14.2 fragile site and renal carcinoma-associated t(3;8) breakpoint, is abnormal in digestive tract cancers. Cell.

[5] Paige AJ (2001). WWOX: a candidate tumor suppressor gene involved in multiple tumor types. Proc. Natl. Acad. Sci. USA.

[6] Gao G, Smith DI (2015). WWOX, large common fragile site genes, and cancer. Exp. Biol. Med. (Maywood).

[7] Tsantoulis PK (2008). Oncogene-induced replication stress preferentially targets common fragile sites in preneoplastic lesions. A genome-wide study. Oncogene.

[8] Debacker K (2007). FRA18C: a new aphidicolin-inducible fragile site on chromosome 18q22, possibly associated with in vivo chromosome breakage. J. Med. Genet..

[9] Bignell GR (2010). Signatures of mutation and selection in the cancer genome. Nature.

[10] Lai LA (2010). Deletion at fragile sites is a common and early event in Barrett’s esophagus. Mol. Cancer Res..

[11] Mashima R, Hishida Y, Tezuka T, Yamanashi Y (2009). The roles of Dok family adapters in immunoreceptor signaling. Immunol. Rev..

[12] Berger AH (2010). Identification of DOK genes as lung tumor suppressors. Nat. Genet..

[13] Küçük C (2015). Global promoter methylation analysis reveals novel candidate tumor suppressor genes in natural killer cell lymphoma. Clin. Cancer Res..

[14] Crowder RJ, Enomoto H, Yang M, Johnson EM, Milbrandt J (2004). Dok-6, a novel p62 Dok family member, promotes Ret-mediated neurite outgrowth. J. Biol. Chem..

[15] Li Wq SL (2010). Downstream of tyrosine kinase/docking protein 6, as a novel substrate of tropomyosin-related kinase C receptor, is involved in neurotrophin 3-mediated neurite outgrowth in mouse cortex neurons. BMC Biol..

[16] Kurotsuchi A (2010). Analysis of DOK-6 function in downstream signaling of RET in human neuroblastoma cells. Cancer Sci..

[17] Jones RB, Gordus A, Krall JA, MacBeath G (2006). A quantitative protein interaction network for the ErbB receptors using protein microarrays. Nature.

[18] Cancer Genome Atlas Research Network (2014). Comprehensive molecular characterization of gastric adenocarcinoma. Nature.

[19] Carter NP (1992). Reverse chromosome painting: a method for the rapid analysis of aberrant chromosomes in clinical cytogenetics. J. Med. Genet..

[20] Croft D (2014). The reactome pathway knowledgebase. Nucleic Acids Res..

[21] Szklarczyk D (2015). STRING v10: protein-protein interaction networks, integrated over the tree of life. Nucleic Acids Res..

[22] Kanehisa, M. *Post-genome Informatics* (Oxford University Press, 2000).

[23] Hu N (2016). Genomic landscape of somatic alterations in esophageal squamous cell carcinoma and gastric cancer. Cancer Res..

[24] Ge H, Liu Z, Church GM, Vidal M (2001). Correlation between transcriptome and interactome mapping data from *Saccharomyces cerevisiae*. Nat. Genet..

[25] D’haeseleer P, Liang S, Somogyi R (2000). Genetic network inference: from co-expression clustering to reverse engineering. Bioinformatics.

[26] Terashima M (2012). Impact of expression of human epidermal growth factor receptors EGFR and ERBB2 on survival in stage II/III gastric cancer. Clin. Cancer Res..

[27] Nagatsuma AK (2015). Expression profiles of HER2, EGFR, MET and FGFR2 in a large cohort of patients with gastric adenocarcinoma. Gastric Cancer.

[28] Betts G (2014). FGFR2, HER2 and cMet in gastric adenocarcinoma:detection, prognostic significance and assessment of downstream pathway activation. Virchows. Arch..

[29] Han N, Kim MA, Lee HS, Kim WH (2015). Evaluation of fibroblast growth factor receptor 2 expression, heterogeneity and clinical significance in gastric cancer. Pathobiology..

[30] Kodama M (2010). Expression of platelet-derived growth factor (PDGF)-B and PDGF-receptor β is associated with lymphatic metastasis in human gastric carcinoma. Cancer Sci..

[31] Jüttner S (2006). Vascular endothelial growth factor-D and its receptor VEGFR-3: two novel independent prognostic markers in gastric adenocarcinoma. J. Clin. Oncol..

[32] Krüttgen A, Schneider I, Weis J (2006). The dark side of the NGF family: neurotrophins in neoplasias. Brain Pathol..

[33] Li Z (2009). High affinity neurotrophin receptors and ligands promote leukemogenesis. Blood.

[34] Zhang Y, Dang C, Ma Q, Shimahara Y (2005). Expression of nerve growth factor receptors and their prognostic value in human pancreatic cancer. Oncol. Rep..

[35] Genevois AL (2013). Dependence receptor TrkC is a putative colon cancer tumor suppressor. Proc. Natl. Acad. Sci. USA.

[36] Pan Y, Zhang J, Liu W, Shu P, Yin B, Yuan J (2013). Dok5 is involved in th signaling pathway of neurotrophin-3 against TrkC-induced apoptosis. Neurosci. Lett..

[37] Ford MG, Valle JD, Soroka CJ, Merchant JL (1997). EGF receptor activation stimulate endogenous gastrin gene expression in canine G cells and human gastric cell cultures. J. Clin. Invest..

[38] Miyazaki Y (1999). Gastrin induces heparin-binding epidermal growth factor-like growth factor in rat gastric epithelial cells transfected with gastrin receptor. Gastroenterology.

[39] Stephens MR (2007). Prognostic significance of gastrin expression in patients undergoing R0 gastrectomy for adenocarcinoma. Gastric Cancer.

[40] Bang YJ (2010). Trastuzumab in combination with chemotherapy versus chemotherapy alone for treatment of HER2-positive advanced gastric or gastro-oesophageal junction cancer (ToGA): a phase 3, open-label, randomized controlled trial. Lancet.

[41] Kim J (2014). Preexisting oncogenic events impact trastuzumab sensitivity in ERBB2-amplified gastroesophageal adenocarcinoma. J. Clin. Invest..

[42] Nam HJ (2012). Evaluation of the antitumor effects and mechanisms of PF00299804, a pan-HER inhibitor, alone or in combination with chemotherapy or targeted agents in gastric cancer. Mol. Cancer. Ther..

[43] Singh R, Kim WJ, Kim PH, Hong HJ (2013). Combined blockade of HER2 and VEGF exerts greater growth inhibition of HER2-overexpressing gastric cancer xenografts than individual blockade. Exp. Mol. Med..

[44] Meulendijks D (2016). Trastuzumab and bevacizumab combined with docetaxel, oxaliplatin and capecitabine as first-line treatment of advanced HER2-positive gastric cancer: a multicenter phase II study. Invest. New Drugs..

[45] Ng BL, Carter NP (2006). Factors affecting flow karyotype resolution. Cytometry. A.

