# Supplementary material for: Chromosomal breaks at FRA18C: association with reduced DOK6 expression, altered oncogenic signaling and increased gastric cancer survival
Source: NPJ Precis Oncol. 2017 May 1;1:9. doi: 10.1038/s41698-017-0012-3 (PMC5859466; doi:10.1038/s41698-017-0012-3)
Supplement: Supplementary file 1 — Supplementary Information [file 41698_2017_12_MOESM1_ESM.docx]

**Supplementary Information**

Figure S1

A


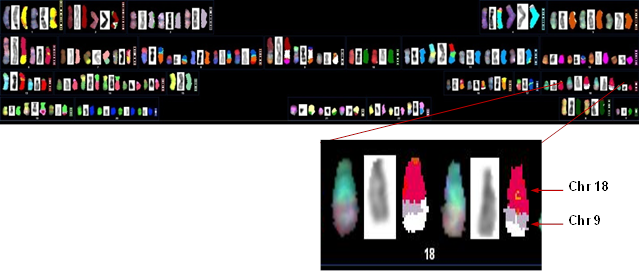


B


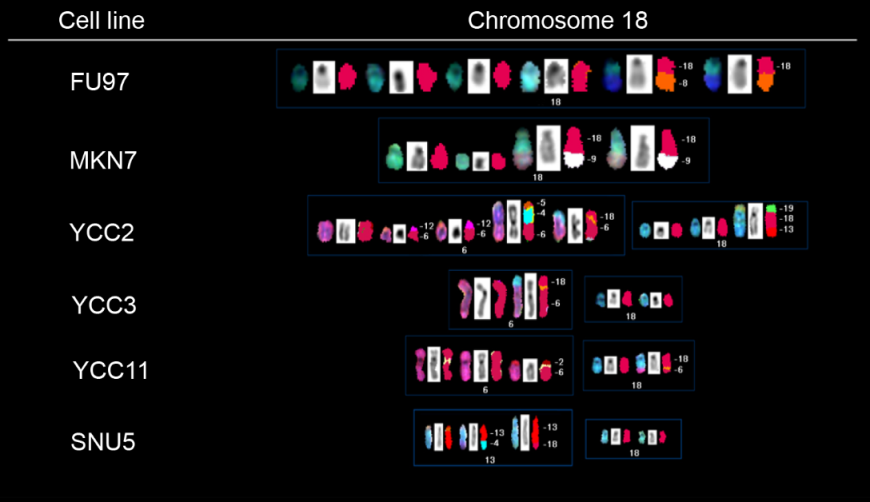


**Fig S1** **Spectral karyotypes of gastric cancer cell lines**

A Spectral karyotype of MKN7 cell line. The recurrent translocated chromosome of interest is enlarged.

B **C**hromosome 18 rearrangements in six gastric cancer cell lines from spectral karyotyping. The rearrangements were: FU97 t(8;18); MKN t(9;18); YCC2 t(6;18); YCC3 t(6;18); YCC11 t(6;18); SNU5 t(13;18).

Figure S2


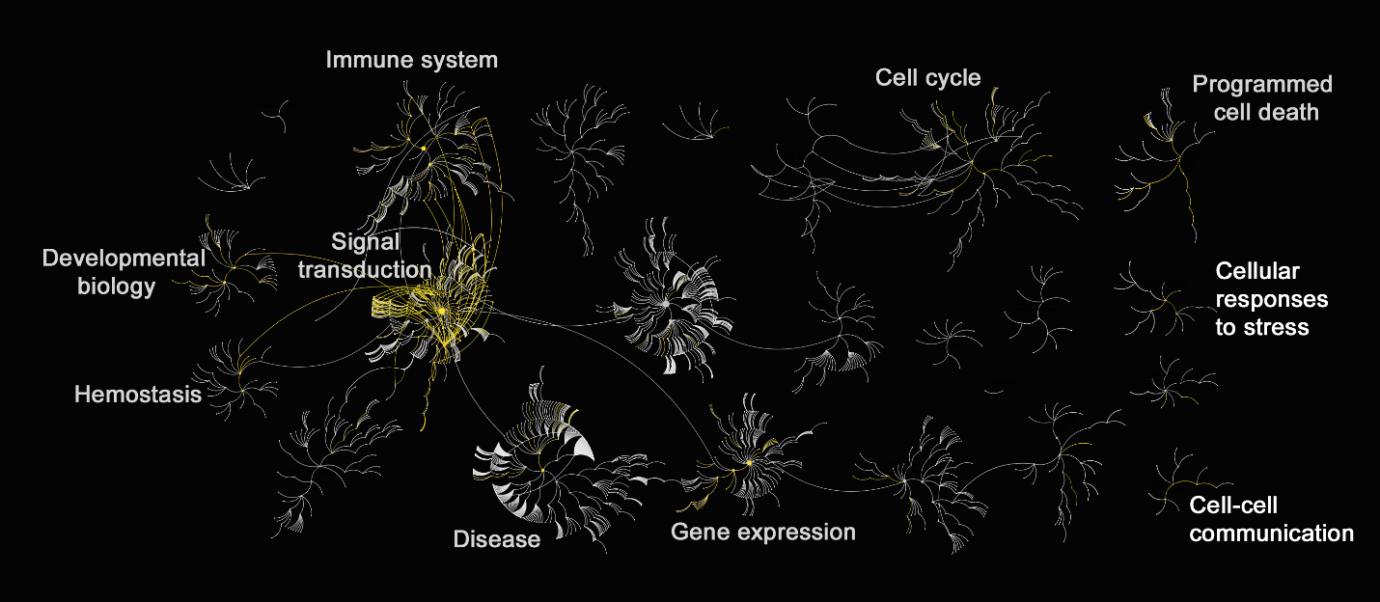


**Fig S2. Ten biological networks enriched in differentially expressed proteins (RPPA data) by Reactome mapping.** The statistically most enriched networks are bright yellow.

Figure S3

**A**





**B**





**Fig S3. Decrease in cell proliferation and colony formation ability after DOK6 knockdown.** YCC16, a gastric cancer cell line with constitutively high DOK6 expression, was stably transfected with a mixture of 4 shRNA constructs; controls were stably transfected with a scrambled shRNA construct (TL304927; OriGene, Rockville, Maryland, USA). (A) Cell proliferation was assayed using the MTS assay by absorbance readings at 490 nm (CellTiter96 AQueous One Solution, Promega, Madison, Wisconsin, USA) on the third day (open bars) and fourth day (hatched bars) after seeding 250 cells in each well of a 6-well plate. (B) Seven hundred and fifty cells were seeded onto 35 mm plates and cultured for 7 days before fixation with 10% buffered formalin for 15 minutes. Colonies were stained with 0.023% crystal violet solution for 1 hour at room temperature and counted. Data in both panels are presented as the average of triplicates and standard deviations.

**Table S1.** PCR primers for *DOK6* exons

RT-PCR

|  | Forward primer | Reverse primer | Size |
| --- | --- | --- | --- |
| Exons 2- 4 | ATTTTCAGACGATGCTGGTTG | CATTCTGTTCCCGCTGCAC | 343 bp |
| Exons 2-5 | ATTTTCAGACGATGCTGGTTG | ATGTGGCCTCTCAGCTCACT | 485 bp |

Genomic PCR

|  | Forward primer | Reverse primer | Size |
| --- | --- | --- | --- |
| Exon 2 | TCTAGCAAAGGACCCAGAAGG | AGTCTTTGGTTTTGCACAGAGG | 275 bp |
| Exon 3 | ATCGAAGACATTTGCCTGTGAG | CAAAGCTTGAACGGAAACCTC | 269 bp |
| Exon 4 | GGAACAGAATGGTAGGTGTGAG | GTTACACCATTGTGGGAAAGTC | 473 bp |
| Exon 5 | TCTTATGCCTACACCAAACCTG | GGACAGCACTCTCTTTCAGGAC | 258 bp |
| Exon 6 | CCATAGCTGAGCAACATGAAAG | ACTCTCTGACATCCAAGGCAAC | 312 bp |
| Exon 7 | CACATCACTCGTCAGAACAGC | GTCAAGTTCTCGTGCCAAATG | 297 bp |
| Exon 8 | TCATCAAGGATCAAGGCAAC | GTAGCTGGGAAATGTCTGTGC | 250 bp |

**Table S2.** Archived primary gastric adenocarcinomas on tissue microarrays

|  | Age | Sex | Lauren  histotype |  |  | Age | Sex | Lauren  histotype |  |  | Age | Sex | Lauren  histotype |
| --- | --- | --- | --- | --- | --- | --- | --- | --- | --- | --- | --- | --- | --- |
| 1 | 76 | F | Diffuse |  | 34 | 78 | M | Intestinal |  | 67 | 77 | M | Intestinal |
| 2 | 70 | M | Intestinal |  | 35 | 50 | M | Intestinal |  | 68 | 63 | M | Intestinal |
| 3 | 75 | M | Mixed |  | 36 | 71 | M | Diffuse |  | 69 | 69 | M | Intestinal |
| 4 | 61 | F | Intestinal |  | 37 | 67 | M | Mixed |  | 70 | 46 | F | Diffuse |
| 5 | 66 | F | Diffuse |  | 38 | 62 | M | Intestinal |  | 71 | 60 | F | Intestinal |
| 6 | 56 | M | Intestinal |  | 39 | 41 | M | Mixed |  | 72 | 72 | M | Intestinal |
| 7 | 67 | M | Intestinal |  | 40 | 75 | F | Diffuse |  | 73 | 51 | F | Diffuse |
| 8 | 48 | M | Intestinal |  | 41 | 61 | M | Intestinal |  | 74 | 80 | F | Mixed |
| 9 | 82 | M | Intestinal |  | 42 | 88 | M | Intestinal |  | 75 | 69 | M | Diffuse |
| 10 | 78 | M | Intestinal |  | 43 | 67 | M | Intestinal |  | 76 | 81 | F | Intestinal |
| 11 | 28 | M | Diffuse |  | 44 | 73 | F | Intestinal |  | 77 | 88 | F | Intestinal |
| 12 | 71 | F | Mixed |  | 45 | 49 | M | Mixed |  | 78 | 64 | M | Mixed |
| 13 | 50 | F | Diffuse |  | 46 | 65 | F | Mixed |  | 79 | 78 | F | Intestinal |
| 14 | 69 | F | Intestinal |  | 47 | 66 | M | Intestinal |  | 80 | 75 | M | Intestinal |
| 15 | 51 | M | Diffuse |  | 48 | 61 | M | Intestinal |  | 81 | 85 | M | Intestinal |
| 16 | 77 | M | Intestinal |  | 49 | 72 | M | Intestinal |  | 82 | 76 | F | Diffuse |
| 17 | 69 | M | Intestinal |  | 50 | 65 | M | Intestinal |  | 83 | 67 | M | Diffuse |
| 18 | 62 | M | Mixed |  | 51 | 51 | F | Diffuse |  | 84 | 48 | M | Intestinal |
| 19 | 72 | M | Intestinal |  | 52 | 75 | F | Intestinal |  | 85 | 76 | M | Intestinal |
| 20 | 35 | F | Diffuse |  | 53 | 78 | M | Intestinal |  | 86 | 79 | M | Intestinal |
| 21 | 73 | M | Intestinal |  | 54 | 63 | M | Diffuse |  | 87 | 73 | F | Intestinal |
| 22 | 86 | F | Diffuse |  | 55 | 63 | F | Diffuse |  | 88 | 67 | F | Intestinal |
| 23 | 78 | F | Diffuse |  | 56 | 66 | F | Intestinal |  | 89 | 62 | M | Diffuse |
| 24 | 74 | F | Intestinal |  | 57 | 75 | M | Intestinal |  | 90 | 60 | F | Diffuse |
| 25 | 44 | M | Diffuse |  | 58 | 53 | M | Diffuse |  | 91 | 52 | F | Intestinal |
| 26 | 69 | M | Mixed |  | 59 | 57 | F | Intestinal |  | 92 | 79 | F | Intestinal |
| 27 | 47 | M | Intestinal |  | 60 | 78 | F | Intestinal |  | 93 | 75 | F | Mixed |
| 28 | 72 | M | Intestinal |  | 61 | 76 | M | Intestinal |  | 94 | 60 | M | Diffuse |
| 29 | 74 | M | Intestinal |  | 62 | 86 | F | Intestinal |  | 95 | 73 | M | Mixed |
| 30 | 73 | F | Intestinal |  | 63 | 72 | M | Mixed |  | 96 | 57 | M | Diffuse |
| 31 | 64 | F | Diffuse |  | 64 | 70 | F | Intestinal |  | 97 | 75 | M | Intestinal |
| 32 | 50 | F | Diffuse |  | 65 | 80 | M | Mixed |  | 98 | 65 | M | Mixed |
| 33 | 66 | F | Intestinal |  | 66 | 82 | M | Intestinal |  | 99 | 74 | F | Mixed |

**Table S3.** Spearman correlation analysis of *DOK6* expression with each of the 34 core signaling genes

*ϒ*_S_ is Spearman’s correlation coefficient. All *P* values are FDR adjusted (0.05).

| Gene | *r_s_* | *P* |  |  | Gene | *r_s_* | *P* |  |  | Gene | *r_s_* | *P* |
| --- | --- | --- | --- | --- | --- | --- | --- | --- | --- | --- | --- | --- |
| Receptors | | |  |  | Ligands | | |  |  | Signaling proteins | | |
| FGFR1 | 0.7451 | 3.49E-69 |  |  | ANGPT1 | 0.6756 | 1.65E-52 |  |  | CAMK2A | 0.4961 | 3.00E-25 |
| GFRA1 | 0.5885 | 3.75E-37 |  |  | FGF1 | 0.6236 | 9.23E-43 |  |  | RASGRF2 | 0.6938 | 2.00E-56 |
| GFRA2 | 0.5644 | 1.11E-33 |  |  | FGF10 | 0.6262 | 3.29E-43 |  |  |  |  |  |
| GFRA4 | 0.3535 | 9.58E-13 |  |  | FGF16 | 0.2929 | 4.95E-09 |  |  | Adaptors | | |
| GRIN2A | 0.5353 | 7.40E-30 |  |  | FGF2 | 0.6371 | 4.11E-45 |  |  | CNKSR2 | 0.6443 | 2.00E-46 |
| IL17RD | 0.4912 | 1.03E-24 |  |  | FGF5 | 0.4191 | 9.18E-18 |  |  | KL | 0.6185 | 6.63E-42 |
| IL5RA | 0.4664 | 3.89E-22 |  |  | FGF6 | 0.2132 | 2.52E-05 |  |  | SHC3 | 0.4378 | 2.06E-19 |
| ITGB3 | 0.6572 | 7.61E-49 |  |  | FGF7 | 0.7380 | 3.03E-67 |  |  |  |  |  |
| KIT | 0.5793 | 8.48E-36 |  |  | GDNF | 0.4697 | 1.82E-22 |  |  | Cell-cell interaction proteins | | |
| RET | 0.5176 | 1.06E-27 |  |  | IL2 | 0.2616 | 1.99E-07 |  |  | NCAM1 | 0.5750 | 3.59E-35 |
| TEK | 0.6935 | 2.32E-56 |  |  | IL5 | 0.2972 | 2.85E-09 |  |  |  |  |  |
|  |  |  |  |  | NRG2 | 0.4624 | 9.61E-22 |  |  | Cytoskeletal proteins | | |
|  |  |  |  |  | NRG3 | 0.5092 | 1.01E-26 |  |  | ACTN2 | 0.5352 | 7.74E-30 |
|  |  |  |  |  |  |  |  |  |  | NEFL | 0.4968 | 2.50E-25 |
|  |  |  |  |  |  |  |  |  |  | SPTA1 | 0.3227 | 9.32E-11 |
|  |  |  |  |  |  |  |  |  |  | SPTBN2 | 0.2038 | 5.73E-05 |

**Table S4.** Differentially expressed proteins from RPPA data

Fold ratio is the ratio of protein expression in bottom quartile/top quartile of *DOK6*-expressing gastric cancers.

*P* values are FDR adjusted (0.05).

RPPA data are from https://www.mdanderson.org/education-and-research/resources-for-professionals/scientific-resources/core-facilities-and-services/functional-proteomics-rppa-core/antibody-lists-protocols/functional-proteomics-reverse-phase-protein-array-core-facility-antibody-lists-and-protocols.html (data freeze March 16^th^, 2016)

Overexpressed proteins

| Gene symbol | Fold ratio | *P* | Array element | Company | Catalog # |
| --- | --- | --- | --- | --- | --- |
| ACACA ACACB | 1.212 | 1.83E-02 | ACC_pS79-R-V | CST | 3661 |
| ARAF | 1.127 | 9.22E-06 | A-Raf-R-V | CST | 4432 |
| ASNS | 1.551 | 1.62E-03 | ASNS-R-V |  |  |
| BCL2L11 | 1.177 | 8.48E-03 | Bim-R-V | Abcam | ab32158 |
| BIRC2 | 1.168 | 4.59E-08 | cIAP-R-V |  |  |
| BRAF | 1.137 | 9.15E-04 | B-Raf_pS445-R-V | CST | 2696 |
| BRD4 | 1.160 | 1.28E-02 | BRD4-R-V | CST | 13440 |
| CCNB1 | 1.601 | 2.67E-07 | Cyclin_B1-R-V | Epitomics | 1495-1 |
| CCNE1 | 1.427 | 9.28E-03 | Cyclin_E1-M-V | Santa Cruz | sc-247 |
| CDH1 | 1.480 | 1.63E-03 | E-Cadherin-R-V | CST | 3195 |
| CLDN7 | 2.057 | 1.81E-05 | Claudin-7-R-V | Novus Biologicals | NB100-91714 |
| CTNNA1 | 1.246 | 2.41E-06 | alpha-Catenin-M-V |  |  |
| CTNNB1 | 1.352 | 3.02E-02 | beta-Catenin-R-V | CST | 9562 |
| DUSP4 | 1.437 | 1.44E-03 | DUSP4-R-V | CST | 5149 |
| EIF4E | 1.185 | 2.67E-07 | eIF4E-R-V | CST | 9742 |
| EIF4EBP1 | 1.392 | 3.88E-07 | 4E-BP1-R-V | CST | 9452 |
| FASN | 1.606 | 8.63E-08 | FASN-R-V | CST | 3180 |
| FOXM1 | 1.207 | 1.44E-03 | FoxM1-R-V | CST | 5436 |
| GSK3A GSK3B | 1.102 | 2.58E-03 | GSK3-alpha-beta-M-V | Santa Cruz | sc-7291 |
| ITGA2 | 1.209 | 4.78E-03 | CD49b-M-V | BD Biosciences | 611016 |
| JAK2 | 1.094 | 3.45E-02 | Jak2-R-V | CST | 3230 |
| MSH2 | 1.288 | 1.32E-05 | MSH2-M-V |  |  |
| MTOR | 1.091 | 4.07E-02 | mTOR-R-V | CST | 2983 |
| PDK1 | 1.085 | 4.66E-03 | PDK1-R-V | CST | 3062 |
| PDK1 | 1.109 | 4.52E-03 | PDK1_pS241-R-V | CST | 3061 |
| PRDX1 | 1.093 | 3.68E-02 | PRDX1-R-V |  |  |
| RB1 | 1.243 | 4.13E-02 | Rb_pS807_S811-R-V | CST | 9308 |
| RPS6KB1 | 1.129 | 1.11E-02 | p70S6K-R-V | Abcam | ab32529 |
| SMAD1 | 1.133 | 2.19E-04 | Smad1-R-V | Abcam | ab33902 |
| SRC | 1.211 | 2.84E-03 | Src-M-V | Millipore | 05-184 |
| SRSF1 | 1.158 | 3.26E-02 | SF2-M-V | Invitrogen | 32-4500 |
| SYK | 1.347 | 7.45E-04 | Syk-M-V | Santa Cruz | sc-1240 |
| TFRC | 1.709 | 6.94E-06 | TFRC-R-V | Novus Biologicals | 22500002 |
| YWHAZ | 1.142 | 3.54E-02 | 14-3-3_zeta-R-V | Santa Cruz | sc-1019 |

Underexpressed proteins

| Gene symbol | Fold ratio | *P* | Array element | Company | Catalog # |
| --- | --- | --- | --- | --- | --- |
| AKT1 AKT2 AKT3 | 0.850 | 6.17E-04 | Akt-R-V | CST | 4691 |
| AKT1 AKT2 AKT3 | 0.861 | 3.26E-02 | Akt_pT308-R-V | CST | 2965 |
| BAD | 0.907 | 1.34E-02 | Bad_pS112-R-V | CST | 9291 |
| BCL2 | 0.840 | 7.78E-05 | Bcl-2-M-V | Dako | M0887 |
| CAV1 | 0.409 | 8.63E-08 | Caveolin-1-R-V | CST | 3238 |
| CCND1 | 0.582 | 7.51E-07 | Cyclin_D1-R-V | Santa Cruz | sc-718 |
| CDH2 | 0.897 | 2.71E-03 | N-Cadherin-R-V | CST | 4061 |
| COL6A1 | 0.730 | 8.63E-08 | Collagen_VI-R-V | Santa Cruz | sc-20649 |
| DPP4 | 0.815 | 5.61E-03 | CD26-R-V | Abcam | ab28340 |
| DVL3 | 0.914 | 5.17E-03 | Dvl3-R-V |  |  |
| EGFR | 0.928 | 6.34E-03 | EGFR_pY1173-R-V | Abcam | ab32578 |
| EIF4EBP1 | 0.761 | 3.34E-06 | 4E-BP1_pS65-R-V | CST | 9456 |
| ESR1 | 0.882 | 4.42E-04 | ER-alpha-R-V | Lab Vision | RM-9101 |
| ETS1 | 0.877 | 4.07E-02 | ETS-1-R-V | Bethyl | A303-501A |
| GSK3A GSK3B | 0.868 | 3.45E-02 | GSK3-alpha-beta_pS21_S9-R-V | CST | 9331 |
| GSK3A GSK3B | 0.844 | 1.32E-02 | GSK3_pS9-R-V |  |  |
| IGF1R | 0.943 | 1.27E-02 | IGF1R_pY1135_Y1136-R-V | CST | 3024 |
| KDR | 0.832 | 4.81E-03 | VEGFR2-R-V | CST | 2479 |
| KIT | 0.633 | 1.63E-12 | c-Kit-R-V | Abcam | ab32363 |
| MYH11 | 0.324 | 1.63E-03 | MYH11-R-V | Novus Biologicals | 21370002 |
| NKX2-1 | 0.551 | 3.02E-02 | TTF1-R-V | Abcam | ab76013 |
| PEA15 | 0.780 | 8.63E-08 | PEA15-R-V | CST | 2780 |
| PEA15 | 0.804 | 3.54E-02 | PEA15_pS116-R-V | Invitrogen | 44-836G |
| PGR | 0.864 | 3.36E-07 | PR-R-V | Abcam | ab32085 |
| PRKCA | 0.798 | 1.23E-03 | PKC-alpha-M-V |  |  |
| PRKCD | 0.907 | 2.73E-03 | PKC-delta_pS664-R-V | Millipore | 07-875 |
| RICTOR | 0.893 | 6.75E-04 | Rictor_pT1135-R-V | CST | 3806 |
| RPTOR | 0.921 | 1.01E-02 | Raptor-R-V | CST | 2280 |
| SMAD3 | 0.934 | 2.09E-02 | Smad3-R-V | Abcam | ab40854 |
| TSC2 | 0.882 | 2.71E-03 | Tuberin_pT1462-R-V | CST | 3617 |
| WWTR1 | 0.883 | 5.22E-05 | TAZ-R-V | CST | 4883 |
| YWHAB | 0.869 | 2.58E-03 | 14-3-3_beta-R-V | Santa Cruz | sc-628 |

**Table S5.** Features of the custom tiling array

|  | | Start* | End* | Interval (bp) | No. of unique features |
| --- | --- | --- | --- | --- | --- |
| **Breakpoint probes** | |  |  |  |  |
| Chr. 9_2MB | | 92,000,000 | 94,000,000 | 2,000,000 | 8,335 |
| Chr. 18_4MB | | 65,500,000 | 69,500,000 | 4,000,000 | 19,048 |
|  | |  |  |  |  |
| **Other probes** | |  |  |  |  |
| Chr. 18 | Positive tiling probes on Chr18 | 50,000,000 | 50,031,000 | 31,500 | 150 |
|  | Positive HD probes on Chr18 | 40,000,000 | 60,000,000 | 20,000,000 | 150 |
| Chr. 9 | Positive tiling Probes on Chr9 | 100,000,000 | 100,031,000 | 31,500 | 150 |
|  | Positive HD Probes on Chr9 | 100,000,000 | 120,000,000 | 20,000,000 | 150 |
| Chr. 1 | Positive tiling Probes on Chr1 | 40,000,000 | 40,031,500 | 31,500 | 150 |
|  | Positive HD Probes on Chr1_150 | 40,000,000 | 60,000,000 | 20,000,000 | 150 |
|  | Positive HD Probes on Chr1_500 | 140,000,000 | 240,000,000 | 100,000,000 | 500 |
| Chr. 7 | Negative tiling Probes on Chr7 | 50,000,000 | 50,031,000 | 31,500 | 150 |
|  | Negative HD Probes on Chr7 | 70,000,000 | 100,000,000 | 30,000,000 | 150 |
| Chr. 4p | Negative tiling Probes on Chr4 | 30,000,000 | 30,031,000 | 31,500 | 150 |
|  | Negative HD Probes on Chr4 | 30,000,000 | 40,000,000 | 10,000,000 | 150 |
|  | |  |  |  |  |
| Normalization and replication probes (Chr. 2) | |  |  |  | 1000 |
| Agilent controls | |  |  |  | 6,539 |

*All base positions refer to reference human genome GRCh37/hg19.

# Table S6. Fosmid and BAC clones used in fission and fusion FISH assays on MKN7 metaphase spreads and primary gastric adenocarcinoma tissues.

| **Fosmid ID** | **Cytoband** | **Start** | **End** | **Label** |
| --- | --- | --- | --- | --- |
| **Fission assay on *DOK6* intron 4** | | | | |
| **G248P89647A7** | 18q22.2 | 67,284,306 | 67,321,278 | SpectrumGreen |
| **G248P800714D5** | 18q22.2 | 67,362,172 | 67,398,114 | SpectrumOrange |
|  | | | | |
| **Fusion assay between chromosome 18 and chromosome 9** | | | | |
| **G248P89647A7** | 18q22.2 | 67,284,306 | 67,321,278 | SpectrumGreen |
| **G248P88925F1** | 9q22.2 | 93,055,252 | 93,095,045 | SpectrumOrange |

| **BAC ID** | **Cytoband** | **Start** | **End** | **Label** |
| --- | --- | --- | --- | --- |
| **Fission assay** | | | | |
| **RP11-22G18** | 18q22.2 | 67,564,184 | 67,743,728 | SpectrumGreen |
| **RP11-98M3** | 18q22.2 | 67,198,763 | 67,355,215 | SpectrumOrange |
|  | | | | |
| **Fusion assay between chromosome 18 and chromosome 9** | | | | |
| **RP11-133K16** | 9q22.2 | 93,540,070 | 93,720,725 | SpectrumGreen |
| **RP11-98M3** | 18q22.2 | 67,198,763 | 67,355,215 | SpectrumOrange |

All base positions refer to reference human genome GRCh37/hg19.
